# FAU-Type Zeolite Synthesis from Clays and Its Use for the Simultaneous Adsorption of Five Divalent Metals from Aqueous Solutions

**DOI:** 10.3390/ma14133738

**Published:** 2021-07-03

**Authors:** Ifeoma V. Joseph, Lubomira Tosheva, Gary Miller, Aidan M. Doyle

**Affiliations:** Department of Natural Sciences, Manchester Metropolitan University, Chester Street, Manchester M1 5GD, UK; g.miller@mmu.ac.uk (G.M.); a.m.doyle@mmu.ac.uk (A.M.D.)

**Keywords:** clay, faujasite, adsorption, nonlinear isotherm, heavy metals, zeolite synthesis

## Abstract

In this research, a vermiculite-kaolinite clay (VK) was used to prepare faujasite zeolites via alkaline fusion and hydrothermal crystallisation. The optimal synthesis conditions were 1 h fusion with NaOH at 800 °C, addition of deionised water to the fused sample at a sample to deionised water mass ratio of 1:5, 68 h of non-agitated ageing of the suspension, and 24 h of hydrothermal treatment at 90 °C. The efficacy of the prepared faujasite was compared to raw clay and a reference zeolite material through adsorption experiments of aqueous solutions containing five divalent cations—Cd, Co, Cu, Pb, and Zn. The results showed that in the presence of competing cations at concentrations of 300 mg L^−1^ and adsorbent loading of 5 g L^−1^, within the first 10 min, about 99% of Pb, 60% of Cu, 58% of Cd, 28% of Zn, and 19% of Co were removed by the faujasite prepared from clay. Two to four parameter nonlinear adsorption isotherms were used to fit the adsorption data and it was found that overall, three and four parameter isotherms had the best fit for the adsorption process.

## 1. Introduction

Clay minerals, ubiquitous to environments with sediments, are phyllosilicates from sedimentary and weathering formations. Two-dimensional layers of tetrahedral (SiO_4_) and octahedral (AlO_6_) structural sheets characterise them. A 1:1 layer arrangement indicates repeating single tetrahedral and octahedral units found in kaolinites, while 2:1 arrangement represents two tetrahedral units for each octahedral unit found in smectites (montmorillonites), vermiculite, and illites [1,2,3]. The reported mineralogy of clays include calcites, smectites, illites, kaolinites, halloysites, quartz, feldspar, and montmorillonite [1,2]. There is an appreciable literature on the use of clays like bentonite [3,4,5,6], kaolinite [7,8,9], montmorillonite [7,8,10], halloysite [11,12], and clay composites [7,13,14] for the removal of heavy metals in aqueous solutions. Due to their abundance in nature, these clays offer cheaper alternatives in purifying water contaminated with heavy metals. Such metals in drinking water can cause debilitating and sometimes terminal conditions.

Clay minerals are predominantly composed of aluminosilicates, which suggests that they may be suitable raw materials for zeolite syntheses. Faujasite (FAU) zeolites, hydrated aluminosilicates used as molecular sieves or ion exchange media [15,16,17], have been prepared for applications ranging from the adsorption of gases [18] to catalysis and the removal of pollutants in water [19,20,21,22,23]. The synthesis of faujasite zeolites has been reported for clays such as illite [23,24], kaolin [25,26], and montmorillonite [24]. The prevalent synthesis method is the pre-alkaline fusion with hydrothermal treatment, factors that influence the synthesis include the alkaline concentration and the ratio of liquid to solid in the precursor [24].

This study investigates the synthesis of faujasite zeolite adsorbents from vermiculite–kaolinite clays via alkaline fusion and hydrothermal treatment and its application in the simultaneous removal of five divalent metals in prepared water samples. The experimental data was modelled with nonlinear adsorption isotherms instead of the linearised forms to avoid the error variance inherent in the latter. Linearisation of adsorption isotherm equations violates the theories and assumptions made in the derivation of adsorption isotherms, hence the need for use of the equations in their nonlinear forms in multisolute adsorption systems.

## 2. Materials and Methods

### 2.1. Materials

The vermiculite-kaolinite clay (VK) was obtained from Anambra, South Eastern Nigeria. This kind of clay has been associated with geophagy, a subset of the psychological disorder called pica [27]. Analytical grades (>99.99% purity) of copper (II) nitrate trihydrate, zinc nitrate hexahydrate, cobalt (II) nitrate hexahydrate, lead (II) nitrate, cadmium (II) nitrate tetrahydrate, and sodium hydroxide were purchased from Sigma Aldrich. Powder X-ray diffraction was undertaken using a Philips X’Pert Powder diffractometer (480 mm diameter, Malvern PANalytical, Almelo, The Netherlands) with a sample spinner and X’Celerator (3.347° active length) 1D-detector in Bragg–Brentano geometry employing copper Kα radiation (Kα1 = 1.5406 Å). An incident beam Soller slit of 0.04 rad, 2° fixed anti scatter slit, incident beam mask of 10 mm, programmable automated divergence slit giving a constant illuminated length of 5.0 mm, and receiving Soller slit of 0.04 rad were used. Data collections from 5 to 120° coupled 2theta/theta at 0.013° step 88 s/step were undertaken. For the raw clay, additional XRD data was collected with orientation in the range 3.5 to 140°, the clay sample was mounted by brushing onto a recess of a zero-background silicon sample holder. All other samples were presented as powders using top-loading sample holders. Data was processed using HighScore Plus version 4.9 (Malvern PANalytical, Almelo, The Netherlands) with phase identification carried out using the Crystallography Open Database implemented within HighScore.

A Carl Zeiss Supra 40VP Scanning Electron Microscope (SEM, Carl Zeiss, Cambridge, United Kingdom) was used to obtain scanning electron micrograph images. The surface areas were obtained with a Micrometrics ASAP 2020 surface area analyser (Micrometrics, Gloucestershire, United Kingdom); samples were degassed for 3 h at 300 °C prior to analysis. The analysis of the chemical composition was obtained with a Rigaku NEX-CG X-ray Fluorescence (XRF, Rigaku Corporation, Tokyo, Japan) instrument. The concentrations of metals in solution were determined using the iCAP 6300 Duo Inductively Coupled Plasma-Optical Emission Spectrometer (ICP-OES, Thermo Scientific, Cambridge, United Kingdom).

### 2.2. Nonlinear Adsorption Isotherms

Nonlinear adsorption model fitting of experimental data was carried out using MS Excel 2016 version 16 solver function for iterative nonlinear least squares regression analysis while nonlinear curve fitting was plotted with Origin^®^ 2019 version 9.6. Modelling of experimental data using the original form of the nonlinear equation maintains the integrity of the data by the avoidance of bias in the use of the linearised form of the equation which is based on an operation on data that has already been transformed leading to errors [28,29]. Nonlinear expressions of two, three, and four parameter adsorption isotherms were used to model the data.

Equilibrium adsorption capacity, *q_e_*, is described by Equation (1):

(1) $$q_{e}=V*\;\frac{\left(C_{i}-C_{e}\right)}{m}$$

where *q_e_* is the equilibrium adsorption capacity (mg g^−1^), *V* the solution volume (L), *m* mass of adsorbents (g), and *C_i_* and *C_e_* the initial and equilibrium concentrations (mg L^−1^), respectively.

The Langmuir isotherm is commonly used to describe homogenous monolayer adsorption on finite adsorption sites without interaction of the layers. The Langmuir adsorption isotherm equation [30] is described in Equation (2):

(2) $$q_{e}=\frac{q_{m}*K_{L}*C_{e}}{1+K_{L}*C_{e}}$$

where *q_e_* is the equilibrium adsorption capacity (mg g^−1^), *q_m_* the maximum adsorption capacity of the adsorbent (mg g^−1^), *C_e_* the equilibrium concentration (mg L^−1^), and *K_L_* the Langmuir isotherm constant (L mg^−1^).

The Freundlich isotherm, with its less restrictive assumption in its empirical model and is applicable to heterogeneous multilayer adsorption [31,32], is described by Equation (3):

(3) $$q_{e}=K_{F}*C_{e}^{1/n}$$

where *q_e_* is the equilibrium adsorption capacity (mg g^−1^), *C_e_* the equilibrium concentration (mg L^−1^), *K_F_* the Freundlich isotherm constant that is an indicator of adsorption, and *n* is a dimensionless parameter that symbolises adsorption density.

The aforementioned isotherms, Langmuir and Freundlich, are two parameter isotherm models. There are more complex models that incorporate more parameters for a better description of the adsorption process, such as three parameter isotherms that include Tóth and Redlich-Peterson, while Fritz-Schlunder IV is an example of a four-parameter model.

The Tóth isotherm is a modification of the Langmuir isotherm for multilayer adsorption. The mathematical expression of Tóth isotherm [33,34] is given in Equation (4):

(4) $$q_{e}=\frac{K_{T}*C_{e}}{{\left(\alpha_{T}+C_{e}\right)}^{1/t}}$$

where *q_e_* is the equilibrium adsorption capacity (mg g^−1^), *C_e_* the equilibrium concentration (mg L^−1^), while *α_T_* and *t* are constants.

The Redlich-Peterson isotherm is an empirical equation resulting from the combination of Freundlich and Langmuir isotherms, it has a versatile applicability for varied concentrations and homogeneity or heterogeneity [31,35,36]. The nonlinear expression is shown in Equation (5):

(5) $$q_{e}=\frac{K_{RP}\;*\;C_{e}}{1+a_{RP}\;*\;C_{e}{}^{b_{RP}}}$$

where *q_e_* is the equilibrium adsorption capacity (mg g^−1^), *C_e_* the equilibrium concentration (mg L^−1^), *b_RP_* is an exponent with values between 0 and 1, while *K_RP_* and *a_RP_* are constants.

The Fritz–Schlunder [35,37] four parameter model, another model based on Freundlich and Langmuir isotherms, is mathematically expressed in Equation (6) as:

(6) $$q_{e}=\frac{\alpha_{1}*C_{e}{}^{\beta_{1}}}{1+\alpha_{2}*C_{e}{}^{\beta_{2}}}$$

where *q_e_* is the equilibrium adsorption capacity (mg g^−1^), *C_e_* the equilibrium concentration (mg L^−1^), while the other parameters are constants.

### 2.3. FAU Zeolite Preparation and Metal Adsorption

**Figure 1:**
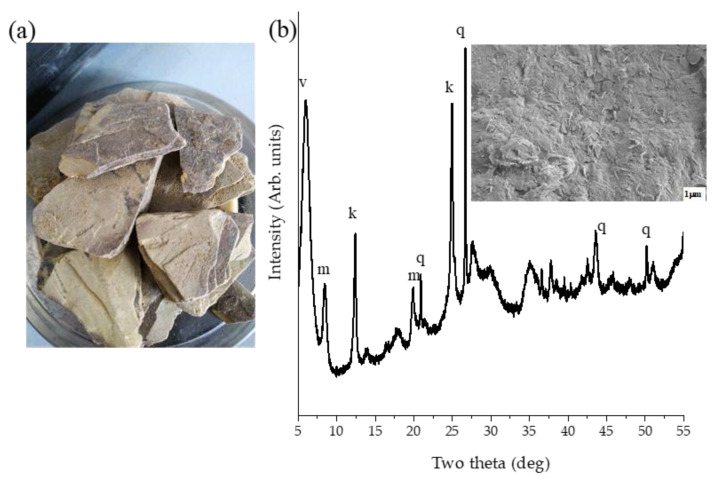
(**a**) Raw vermiculite-kaolinite clay (VK) and (**b**) phase identification and micrograph of VK.k kaolinite, m muscovite, v vermiculite, and q quartz. Insert contains an SEM image.

The hydrothermal synthesis with a prior alkaline fusion method used was based on a modification of the method reported by Shigemoto et al. [38,39]. VK samples were bulky (Figure 1a) and were first crushed and sieved to particle sizes below 150 µm before any of the synthesis steps. The clays were used with and without pretreatment; pretreatment involved thermal activation and acid leaching (cf. Section 3.3). Appropriate amounts of the crushed samples were mixed with NaOH at a VK to NaOH mass ratio of 1:1.2 and fused at 600 °C or 800 °C for 1 to 4 h. Predetermined amounts of fused samples were mixed with deionised water at mass ratios of 1:5, 1:10 and 1:15 in polypropylene reactors and aged at room temperature for 0 to 72 h. Aged samples were hydrothermally treated at 90 °C for 0 to 72 h.

A reference faujasite zeolite, ZRef-FAU, was prepared via the method described by Valtchev et al. [40] with BET (Brunauer Emmett Teller) specific surface area of 626 m^2^ g^−1^ and Si/Al of 2.3.

Batch adsorption studies were carried out in triplicates at 25 °C with adsorbent loading from 2.5 to 20 g L^−1^. Stock and standard solutions of the five metal salts in concentration range of 100 to 500 mg L^−1^ were prepared by the addition of an appropriate amount of each metal salt to deionized water in a volumetric flask [39]. The appropriate amount of adsorbent was added to 20 mL of the aqueous solution in separate 50 mL polypropylene bottles. The samples were agitated using a Gerhardt Laboshake for 0 to 180 min, filtered by centrifugation for 3 min at 3800 rpm, and analysed by ICP-OES. The performance of the optimal CAN zeolite sample, ZVK–FAU, was tested for adsorbent loadings of 5 to 15 g L^−1^ at 90 min.

## 3. Results and Discussion

### 3.1. Clay Characterisation

Figure 1a shows the image of the raw clay prior to crushing and sieving. The mineralogy of the raw sample, measured by XRD, showed that the major phases present were vermiculite, kaolinite, muscovite, and quartz as shown on Figure 1b. Figure S1 shows the XRD patterns of the oriented clay. The accuracy of the collected data was verified by the location of the quartz (100) peak at 20.828° 2θ (expected value 20.865° 2θ). The phases were assigned based on the overall match score calculated from the peak positions and profiles with additional verification based on the positions of low angle peaks. For muscovite, these are the peaks at 3.935° 2θ (d = 22.455 Å) (001) shown in Figure S1 and 8.352° 2θ (d = 10.587° 2θ) (002) shown in Figure 1 and Figure S1; for vermiculite the peak at 5.918° 2θ (d = 14.933 Å) (001) and for kaolinite the peak at 12.304° 2θ (d = 7.194 Å) (002). Vermiculites are among 2:1 type clays while kaolinites are 1:1 types in terms of the sandwiching of tetrahedral and octahedral sheets. The SEM image on Figure 1b shows the micrograph taken after crushing and sieving the raw clay to <150 microns. The particle shapes were of irregular plates with some flaky needle-like particles on the surface.

Table 1 shows the XRF analysis of raw VK used in this study. The material was mainly composed of SiO_2_, Al_2_O_3_, Fe_2_O_3_, MgO, and TiO_2_ with Si/Al mass ratio of 2.9. The BET specific surface area was 95 m^2^ g^−1^ and a micropore volume of 0.009 cm^3^ g^−1^ was determined.

### 3.2. VK as Adsorbent

**Figure 2:**
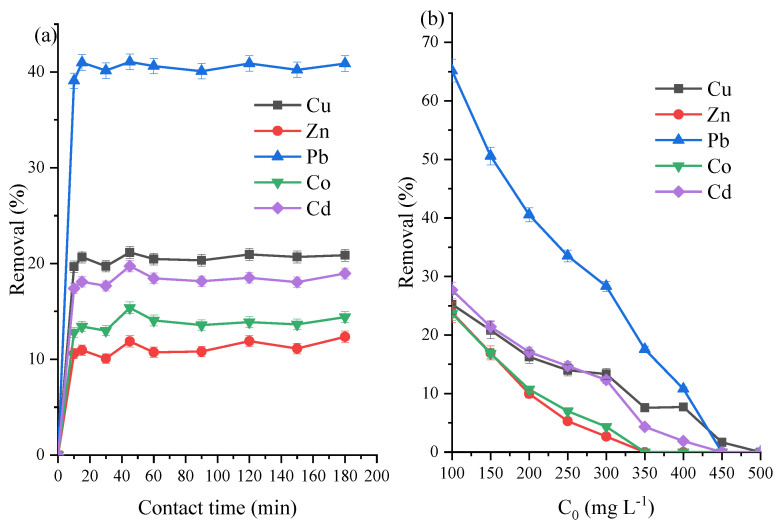
Raw VK adsorption studies using 5 g L^−1^ clay loading for (**a**) *C*_0_ = 200 mg L^−1^ for 10 to 180 min and (**b**) *C*_0_ = 100 to 500 mg L^−1^ at 90 min.

Simultaneous adsorption experiments for aqueous solutions containing Cd, Co, Cu, Pb, and Zn using the raw VK as adsorbent are presented in Figure 2a,b and Table 2. The % removal efficiency (% removal) was calculated using Equation (7):

(7) $$\%\;Removal=\left(\frac{C_{i}-C_{e}}{C_{i}}\right)*100$$

*C_i_* is the initial concentration while *C_e_* is the equilibrium concentration with units of mg L^−1^.

An optimal adsorption duration of 90 min was selected due to the results of the adsorption kinetics at 200 mg L^−1^ from *t* = 0 to *t* = 180 min (Figure 2a) and adsorbent load of 5 g L^−1^. The five heavy metals had their maximum adsorption at the lowest concentration (100 mg L^−1^) with the trend Pb > Cd > Cu > Co = Zn (Figure 2b) at VK loading of 5 g L^−1^. As shown on Table 2 at 200 mg L^−1^ and 90 min, doubling and tripling the amounts of raw VK used as adsorbents resulted in approximately proportional increments in all metals except Pb, which showed a more sedate increment.

### 3.3. Zeolite Characterisation

**Figure 3:**
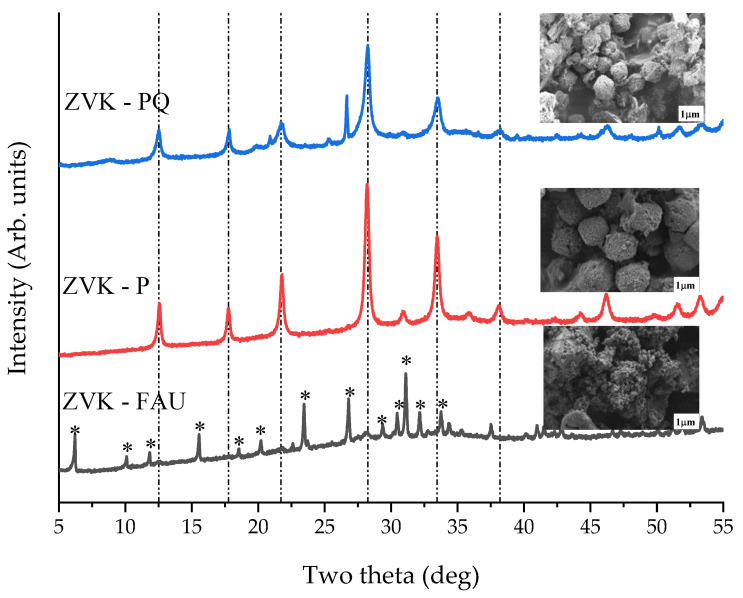
XRD patterns of zeolites from VK with no pretreatment (ZVK-FAU) and pretreatments via thermal activation (ZVK-P) and acid leaching (ZVK-PQ). Dashed lines represent the main GIS-NaP1 peaks, * FAU peaks. Inserts contain SEM images of the corresponding samples.

Zeolite synthesis from VK via alkaline fusion and hydrothermal route [38,41,42] was initially carried out without pre-treatment of the raw VK, with thermal activation at 800 °C for 1 h, and acid leaching using 1:5 VK to HCl (5 M) refluxed at 90 °C for 3 h. NaOH and VK fusion was at 600 °C for 4 h, fused sample to deionised water ratio of 1:10, ageing at 68 h, and hydrothermal treatment at 90 °C for 24 h. Figure 3 shows the XRD patterns and SEM images of the samples prepared using the three pre-treatment conditions. Without pre-treatment, the synthesis yielded a crystalline phase that was predominantly FAU zeolite (ZVK-FAU). Thermal activation yielded a single phase of gismondine (GIS-NaP1) zeolite (ZVK-P), while synthesis with acid leached pre-treatment sample resulted in a mixed phase of NaP1 and quartz (ZVK-PQ).

Thus, synthesis with no prior treatment of the raw VK for FAU zeolite was selected for further experiments. To obtain optimal synthesis parameters for FAU zeolite, variations were made in the duration of ageing, extent of hydrothermal treatment, amount of deionised water, NaOH fusion temperature, and agitation during ageing.

**Figure 4:**
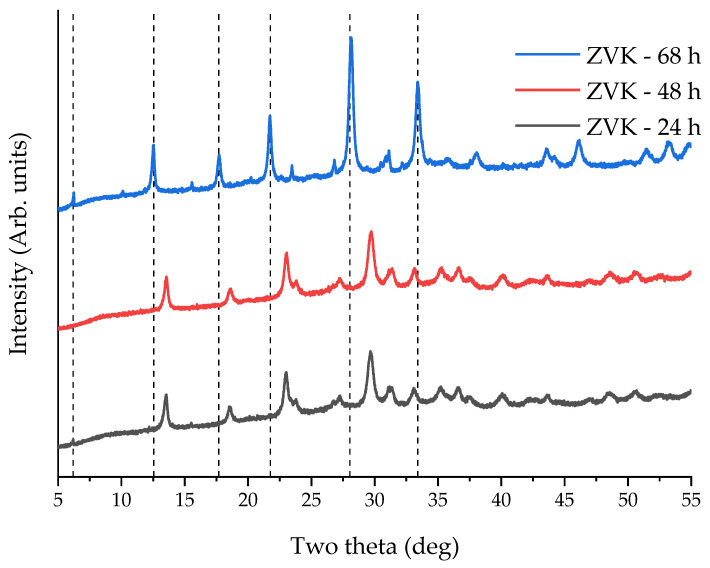
XRD patterns of FAU zeolite from VK for 24, 48, and 68 h ageing at 90 °C for 24 h. Dashed lines represent the main FAU zeolite peaks.

With ageing duration variations for 24 to 68 h, the results are shown in Figure 4. The 68 h ageing resulted in the crystallisation of FAU zeolite. The 24 h, 48 h, and 68 h samples had BET specific surface areas of 60 m^2^ g^−1^, 61 m^2^ g^−1^, and 219 m^2^ g^−1^, respectively, indicating a distinctive structural transition between 48 and 68 h.

At a hydrothermal treatment temperature of 90 °C and 68 h ageing (Supplementary Information Figure S2), the extent of crystallisation was tested for 0, 12, 24, 36, 48, and 72 h and the BET specific surface areas obtained were 52, 81, 219, 108, 33, and 26 m^2^ g^−1^, respectively. From Supplementary Information Figure S2, the 0 HT sample (prepared without hydrothermal treatment) resulted in a predominantly amorphous phase as expected since alkaline fusion leads to the dissolution of aluminosilicate crystalline phases [43]. At 12 h hydrothermal treatment (12 HT), the presence of FAU-type zeolite can be seen in the XRD pattern, the amount of which increases as the duration is extended to 24 h. The BET specific surface areas of the latter two samples increased from 81 to 219 m^2^ g^−1^. Hydrothermal treatment for 36 h showed more distinct FAU zeolite morphology but the specific surface area was reduced to 108 m^2^ g^−1^ while for 48 and 72 h HT, the crystallinity became less defined with a similar trend in the reduction of the specific surface areas observed (Supplementary Information Figure S2). Thus, an optimal hydrothermal time of 24 h was selected.

Water plays a critical role in the hydrothermal synthesis of FAU zeolites, this includes silica depolymerisation, structure directing agent in the pre-nucleation of zeolite, and in the dissolution of crystalline phases when mixed with an alkaline compound [44,45,46]. Mora-Fonz et al. [44] found that in solution, the bond strength of interspecies interaction is in the order of silicate-silicate> silicate-water > water-water. In hydrothermal synthesis of zeolites, these interactions dictate the extent in which silicates aggregate in solution [44]. Hydrophobicity and hydrophilicity of the raw material affect the interaction of the silicate species with water molecules. Supplementary Information Figure S3a, shows the effects of changing the fused sample to deionised water ratios from 1:5 to 1:10 and 1:15. Superior crystallinity and phase purity was obtained using the lower amount of deionised water (1:5); beyond 1:10, the product was predominantly amorphous as shown by the dashed lines for major FAU zeolite peaks in Supplementary Information Figure S3a. The more defined crystallinity with the lowest water ratio could be as a result of the interaction of the hydrated species and the water affinity of the clay material which favoured the nucleation of FAU zeolites. The BET specific surface area of FAU zeolite obtained from 1:5 ratio was 307 m^2^ g^−1^ while that of 1:15 was 57 m^2^ g^−1^.

With an optimal deionised water content of 1:5 for FAU synthesis from VK, the alkali fusion temperature and duration were then varied. From the initial trials at 600 °C and 4 h duration, the fusion of VK with NaOH was done at the same temperature but a lower duration (1 h). The XRD patterns of that is shown in Supplementary Information Figure S3b where 600 °C fusion of NaOH with VK for an hour showed more pronounced XRD patterns and provides 3 h energy savings. Hence, 1 h alkali fusion meets the condition for FAU zeolite from VK with high crystallinity. Supplementary Information Figure S3b also shows the effect of increasing the temperature from 600 °C to 800 °C for the same 1 h duration. The specific surface area measurements indicated that the FAU zeolite prepared using fusion at 600 °C for 1 h sample had a specific surface area of 300 m^2^ g^−1^, while that of the 800 °C 1 h sample had a specific surface area of 303 m^2^ g^−1^.

**Figure 5:**
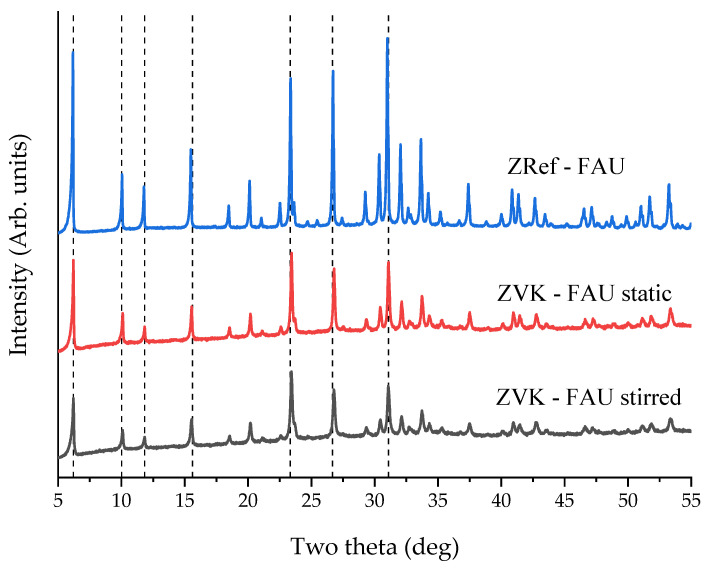
XRD patterns of FAU zeolites from VK (ZVK-FAU) under stirred and static ageing. Dashed lines represent main FAU zeolite peaks.

All the above results were obtained with samples that were stirred during ageing. A comparison of FAU zeolites obtained from samples that were stirred and samples that were only stirred for 5 min and then left static for ageing prior to hydrothermal treatment is presented in Figure 5. The FAU zeolites were obtained with these parameters: 800 °C fusion for 1 h, 1:5 addition of deionised water, ageing (agitated or static) for 68 h, and hydrothermal treatment at 90 °C for 24 h. As can be seen in Figure 5, the crystallinity is somewhat superior for static ageing relative to the same duration with agitated ageing. The static ageing sample, termed ZVK-FAU, was used for the adsorption experiments.

The XRF chemical composition of the optimal adsorbent, ZVK-FAU and the reference zeolite, ZRef-FAU is shown in Table S2. The Si/Al ratio of the reference material and the prepared zeolite was 1.9, the Na contents were similar, but ZVK-FAU had more Fe and Ca than ZRef-FAU.

### 3.4. ZVK-FAU Removal Efficiency

**Figure 6:**
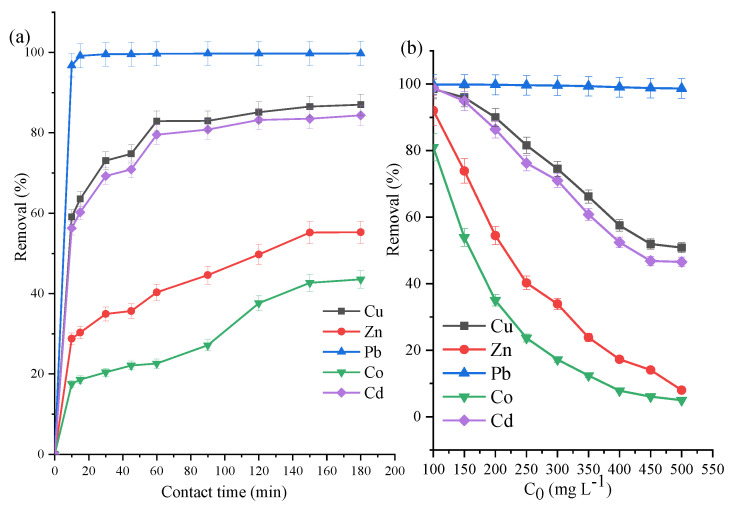
FAU from VK (ZVK-FAU) simultaneous removal of five metals at (**a**) 300 mg L^−1^ from 0 to 180 min and (**b**) 90 min for *C*_0_ = 100 to 500 mg L^−1^ and 5 g L^−1^ zeolite loading.

Firstly, the simultaneous adsorption of Cd, Co, Cu, Pb, and Zn was studied using the optimised zeolite sample (ZVK-FAU) to determine the optimal duration for adsorption. At a concentration of 300 mg L^−1^ for each of the five metals with adsorbent loading of 5 g L^−1^, aliquots of the solution were taken for separation via centrifugation at 0, 10, 20, 30, 40, 60, 90, 120, 150, and 180 min. The % removal, i.e., the amount of heavy metal removed for the specified duration, was calculated using Equation (1) and plotted in Figure 6a.

From Figure 6a and Figure S4, the three most adsorbed metals (Pb, Cu, and Cd) showed no appreciable increase beyond 60 min while the adsorption of Zn and Co slightly increased. An optimal time of 90 min was selected to allow the metals achieve equilibrium in adsorption. In varying the adsorbent loading at 2.5, 5, 10, 15, and 20 g L^−1^, the simultaneous adsorption using a concentration of 300 mg L^−1^ is shown in Table 3. From the results in this Table, it can be seen that with a competitive adsorption of five divalent cations, 98.15% of Pb was removed even at a ZVK-FAU loading of 2.5 g L^−1^. 37.7% Cu, 35.7% Cd, 12.5% Zn, and 6.7% Co were also removed at that adsorbent loading. Doubling the initial loading also doubled the amounts removed for Cu and Cd while Zn and Co removal increased by 21.4% and 13.7%, respectively. The loading had a proportional relationship with the amounts of metals removed—the higher the loading, the higher the % removal of the five divalent metals.

At 90 min and 5 g L^−1^ adsorbent load, the plot for the simultaneous adsorption at 100–500 mg L^−1^ is shown in Figure 6b with the trend Pb > Cu > Cd > Zn > Co for all concentrations. For the untreated clay (Figure 2), the prepared zeolite (Figure 6), and the reference zeolite reported by Joseph et al. [39], there was a preferential uptake of Pb over the other four metals competing for adsorption sites. This could be as a result of the effect of ionic radii and electronegativity of the metals [39,47,48].

There is a marked improvement in the % removal when compared with that of raw clay (VK) shown in Figure 2. For instance, even in the presence of four other competing metals, Pb had about 100% removal using ZVK-FAU while the raw VK had less than 65% removal at the lowest concentration, which progressively lowered as the concentration increased. Using the prepared FAU zeolite (ZVK-FAU) as adsorbents, all of the five heavy metals had much higher amounts removed in comparison with the raw VK adsorbent. Adsorption experiments with the reference adsorbent, ZRef-FAU, has been reported by Joseph et al. [39] with a selectivity trend of Pb > Cd > Cu > Zn > Co. It can be seen that there exists a higher selectivity of Cu over Cd in ZVK-FAU compared to Cd over Cu in ZRef-FAU [39]. Even though the two adsorbents had similar faujasite structures, there were factors that could account for the disparity in adsorption preferences and quantities adsorbed. These factors might include the composition and specific surface area of both adsorbents—one made from pure components, the other prepared from an impure one.

### 3.5. ZVK-FAU Nonlinear Adsorption Isotherms

**Figure 7:**
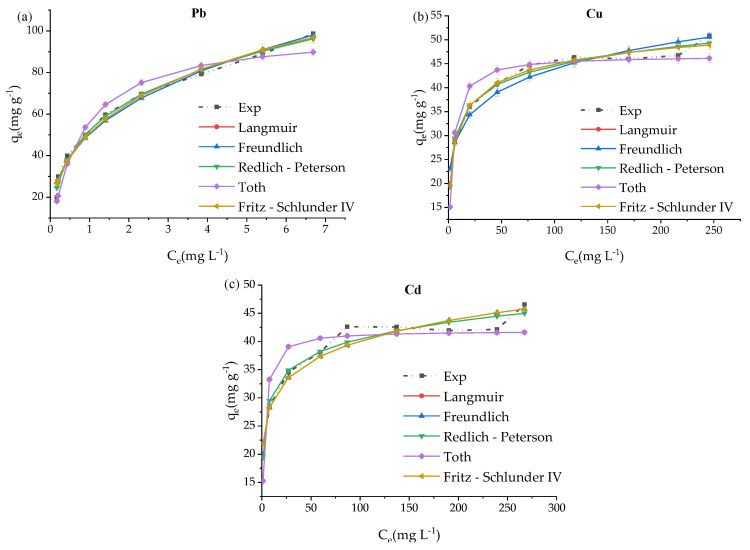
ZVK-FAU nonlinear adsorption isotherms plots for the simultaneous removal of five metals at 90 min, *C*_0_ = 100 to 500 mg L^−1^ and 5 g L^−1^ zeolite loading for (**a**) Pb, (**b**) Cu, and (**c**) Cd.

**Figure 8:**
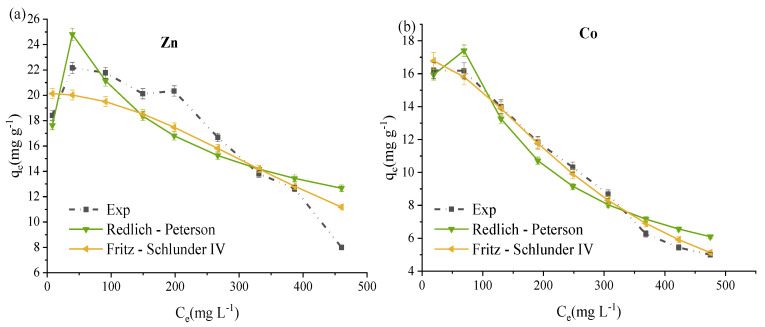
ZVK-FAU nonlinear adsorption isotherms plots for the simultaneous removal of five metals at 90 min, *C*_0_ = 100 to 500 mg L^−1^ and 5 g L ^−1^ zeolite loading for (**a**) Zn and (**b**) Co.

Nonlinear isotherm curve fittings are shown in Figure 7 and Figure 8 for five metals simultaneous adsorption using the prepared ZVK-FAU as adsorbent. The most adsorbed metals (Pb, Cu, and Cd) were reasonably fitted to Langmuir, Freundlich, Redlich-Peterson, Tóth, and Fritz-Schlunder IV adsorption isotherms as shown in Figure 8, the dot dash lines represent experimental data (Exp on the legend). The least adsorbed metals, Co and Zn, could only be fitted to Redlich-Peterson and Fritz-Schlunder IV adsorption isotherms represented in Figure 8. The empirical model parameters for the five metals regarding the plots of Figure 7 and Figure 8 are tabulated in Table 4. All the data for the empirical model fittings were from the competitive and simultaneous adsorption of the five metals (Pb, Cu, Cd, Zn, and Co) with initial metal concentrations of 100 to 500 mg L^−1^. The criterion for selecting the adsorption isotherm model is one in which the Pearson’s coefficient of regression squared (*R*^2^) is as close to unity as possible.

For Pb, the maximum amount adsorbed, *q*_max_, was 98.6 mg g^−1^ from the experimental data. A nonlinear regression analysis using Langmuir isotherm gave a 100.1 mg g^−1^ with a 0.957 Pearson’s coefficient of regression squared (*R*^2^) and 1.3 L g^−1^ as the Langmuir constant, *K_L_* (Table 4 and Figure 7). Fittings using Freundlich, Redlich-Peterson, Tóth, and Fritz-Schlunder IV indicates that all can describe the adsorption isotherm of Pb on faujasite zeolite from vermiculite-kaolinite clay. Visual inspection of Figure 7 and the accompanying parametric data on Table 4 revealed Tóth isotherm as the least best fit among the five isotherms tested. The trend for the five isotherms are: Redlich-Peterson > Fritz-Schlunder IV > Freundlich > Langmuir > Tóth. As can be seen in Figure 6 and Figure 7, Pb was preferentially adsorbed even in the presence of four competing cations and it can be inferred that even at a considerably higher concentration, the selectivity would still favour the adsorption of Pb. The Redlich-Peterson model best described the adsorption process in which monolayer and multilayer adsorption are possibilities for the faujasite from VK used in this study.

The experimental data for the adsorption of Cu had *q*_max_ of 50.8 mg g^−1^ while Langmuir fitting gave 46.7 mg g^−1^ with *R*^2^ of 0.915 (Table 4). The nonlinear model fitting trend as shown in Figure 7 and Table 4 is: Fritz-Schlunder IV > Redlich-Peterson > Freundlich > Langmuir > Tóth. There was not much difference between the fitting for Fritz-Schlunder IV and Redlich-Peterson models especially at lower concentrations, the difference between their *R*^2^, 0.001, is minimal thereby offering a flexibility in the choice of the best model between the two.

For Cd, 46.5 mg g^−1^ was the *q*_max_ from the experiment while the Langmuir model gave 41.9 mg g^−1^ with *R*^2^ of 0.836 as shown on Table 4. Figure 7 and Table 4 show that the trend is: Redlich-Peterson > Fritz-Schlunder IV > Freundlich > Langmuir > Tóth. For all three metals, Freundlich was an improvement over Langmuir indicating monolayer adsorption since one of the main assumptions for Langmuir empirical derivation was based on monolayer adsorption.

Zn and Co, the least adsorbed among the five metals, had experimental *q*_max_ values of 22.2 mg g^−1^ and 16.2 mg g^−1^, respectively. Only two out of the five models, Redlich-Peterson and Fritz-Schlunder IV, were able to fit the data obtained for the two least adsorbed metals. For both Zn and Co, Fritz-Schlunder IV was the best fit with *R*^2^ of 0.8110 and 0.9907, respectively, as shown in Figure 8 and Table 4.

In comparison, Table 5 shows the full nonlinear isotherm modelling parameters for the simultaneous adsorption of five metals using reference sample ZRef-FAU. Similarly, Pb, Cu, and Cd were the most adsorbed while Zn and Co the least adsorbed. The experimental values of *q*_max_ for Pb, Cd, Cu, Zn, and Co were 99.7 mg g^−1^, 77.0 mg g^−1^, 61.4 mg g^−1^, 37.7 mg g^−1^, and 29.4 mg g^−1^, respectively. The empirical model fitting trends (Table 5) for Pb were: Freundlich > Redlich-Peterson > Fritz-Schlunder IV > Langmuir > Tóth, Cd: Redlich-Peterson > Freundlich > Tóth > Fritz-Schlunder IV > Langmuir, Cu: Redlich-Peterson > Freundlich > Fritz-Schlunder IV > Langmuir > Tóth, Zn: Redlich-Peterson > Langmuir > Fritz-Schlunder IV > Freundlich > Tóth, and Co: Langmuir > Fritz-Schlunder IV > Redlich-Peterson > Freundlich > Tóth.

The three parameter Redlich-Peterson and the four parameter Fritz-Schlunder IV adsorption isotherms resulted from the modification of the Langmuir and the Freundlich isotherms, while three parameter Tóth isotherm is a modification of the Langmuir isotherm. This implies that for both prepared and reference FAU zeolites, a best fit where *R*^2^ is closer to unity for either Redlich-Peterson or Fritz-Schlunder IV is a confirmation for Freundlich isotherm if the *R*^2^ is greater than that of Langmuir isotherm. That is the case for Cd, Cu, and Pb for the two zeolites tested. On the other hand, the Langmuir isotherm is probably a better fit for Co and Zn. This subtle distinction can be lost when linearised forms of adsorption isotherms are used. For instance, a study by Joseph et.al [39] in which the linearised forms of adsorption isotherms were used, proposed Langmuir isotherm in the simultaneous adsorption of Cd, Co, Cu, Pb, and Zn. Nebaghe et al. [49] compared the linear and non-linear forms of isotherm equations that included Freundlich, Langmuir, Redlich-Peterson and Fritz-Schlunder IV for the adsorption of Cu in a single solute system. They found that the non-linear forms of the equations gave a better representation of the equilibrium isotherm for Cu where Fritz-Schlunder IV provided the best fit. The adsorption isotherms for Pb, Cu and Mn in a three solute system was best fitted with non-linear Freundlich equation as reported by Zand et al. [50]. Based on the Freundlich isotherm for this research, the most adsorbed metals (Pb, Cd and Cu) occupied the exponentially distributed active sites most likely by virtue of their hydrated ionic radii and electronegative attraction to the binding sites [36,39,47,48]. Meanwhile for the least adsorbed metals, Zn and Co, the remaining vacant binding sites likely behaved as monolayers where the intermolecular attractive forces are progressively reduced [36].

For both ZVK-FAU and ZRef-FAU adsorbents, the nonlinear adsorption isotherm fittings indicated possible heterogeneous multi-layered adsorption with a high likelihood of layer interactions as seen by the generally higher fit of the other isotherms in comparison to the Langmuir isotherm [31,32].

## 4. Conclusions

The elemental composition and mineralogy of the clay used in this study was determined using XRF, and XRD. This clay was used to prepare faujasite (FAU) zeolites using alkali fusion and hydrothermal treatment methods. The synthesis parameters were optimised by varying the alkali fusion temperature and duration, the amount of deionised water added to the fused material, the duration of ageing and hydrothermal treatment as well as the use of agitation during ageing. The simultaneous adsorption of five divalent metals (Cd, Co, Cu, Pb, and Zn) from aqueous solutions using the vermiculite-kaolinite clay (VK), the FAU zeolite prepared from the clay (ZVK-FAU) and a reference FAU zeolite (ZRef-FAU) was studied in batch experiments. The results showed that the performance of the prepared adsorbent was comparable to the reference faujasite zeolite sample with a high selectivity for Pb in the presence of the four other competing cations. For the three adsorbents, Pb had the highest amounts adsorbed (*q*_max_). For ZVK-FAU, the three most adsorbed divalent metals (Pb, Cu, and Cd) were best described by Redlich-Peterson nonlinear isotherm, while the least adsorbed (Zn and Co) were best described by Fritz-Schlunder four parameter isotherms. On the other hand, ZRef-FAU was mainly Redlich-Peterson and Freundlich for the four most adsorbed metals (Pb, Cu, Cd, and Zn). The FAU zeolite prepared from clay significantly improved the adsorption capacity towards heavy metals compared to the unmodified clay.

## Figures and Tables

**Table 1:** XRF chemical compositions (wt%) of raw VK.

MgO	Al_2_O_3_	SiO_2_	K_2_O	CaO	TiO_2_	MnO	Fe_2_O_3_
3.18	15.10	43.90	0.77	0.66	0.98	0.12	5.91

**Table 2:** Effect of raw VK loading variation (*C*_0_ = 200 mg L^−1^ at 90 min) on the percentage removal (%) of divalent metals.

VK Load (g L^−1^)	Cu (%)	Zn (%)	Pb (%)	Co (%)	Cd (%)
5.0	16.3	10.0	40.5	10.7	17.1
10.0	30.9	18.1	61.2	20.2	23.9
15.0	43.6	28.2	75.9	29.6	32.4

**Table 3:** Effect of FAU zeolite from VK (ZVK-FAU) loading variation (*C*_0_ = 300 mg L^−1^ at 90 min) on the percentage removal (%) of divalent metals.

ZVK-FAU Load (g L^−1^)	Cu (%)	Zn (%)	Pb (%)	Co (%)	Cd (%)
2.5	37.7	12.5	98.1	6.7	35.7
5.0	74.5	33.9	99.5	20.4	71.0
10.0	97.3	79.7	99.9	59.6	97.2
15.0	99.4	97.0	99.9	90.5	99.7
20.0	99.7	99.4	100.0	97.8	99.9

**Table 4:** ZVK-FAU nonlinear adsorption isotherms (*C*_0_ = 100 to 500 mg L^−1^ at 90 min and 5 g L^−1^ adsorbent loading).

Model	Parameter	Pb	Cu	Cd	Zn	Co
Langmuir	*q*_max_ (mg g^−1^)	100.153	46.712	41.924	n/a	n/a
	*K_L_* (L mg^−1^)	1.292	0.314	0.502	n/a	n/a
	*R* ^2^	0.957	0.915	0.836	n/a	n/a
Freundlich	1/*n*	0.349	0.154	0.135	n/a	n/a
	*K_F_*	50.521	21.615	21.515	n/a	n/a
	*R* ^2^	0.986	0.956	0.948	n/a	n/a
Redlich-Peterson	*K_RP_*	425.625	37.795	72.577	3.281	1.216
	*a_RP_*	7.127	1.349	2.884	0.029	0.003
	*b_RP_*	0.731	0.896	0.896	1.356	1.659
	*R* ^2^	0.992	0.986	0.967	0.736	0.946
Tóth	*K_T_*	6.734	4.599	15.123	n/a	n/a
	α_*T*_	0.774	3.182	1.993	n/a	n/a
	*t*	14.872	10.157	2.772	n/a	n/a
	*R* ^2^	0.953	0.907	0.829	n/a	n/a
Fritz-Schlunder IV	β_1_	0.3687	0.5122	0.1351	0.0005	0.0001
	β_2_	1.4019	0.4816	0.0001	1.9657	1.8211
	α_1_	51.2060	27.5655	21.5237	20.1108	16.8901
	α_2_	0.0046	0.5964	0.0004	0.0000	0.0000
	*R* ^2^	0.9877	0.9878	0.9484	0.8110	0.9907

**Table 5:** ZRef-FAU nonlinear adsorption isotherms (*C*_0_ = 100 to 500 mg L^−1^ at 90 min and 5 g L^−1^ adsorbent loading).

Model	Parameters	Pb	Cu	Cd	Zn	Co
Langmuir	*q*_max_ (mg g^−1^)	111.081	46.874	52.105	34.820	27.098
	*K_L_* (L mg^−1^)	3.835	1.991	6.353	3.988	3.761
	*R* ^2^	0.977	0.904	0.941	0.891	0.988
Freundlich	1/*n*	0.617	0.180	0.198	0.088	0.031
	*K_F_*	128.232	26.921	37.056	24.687	23.048
	*R* ^2^	0.992	0.991	0.993	0.849	0.336
Redlich-Peterson	*K_RP_*	4228.923	738.597	1587.369	206.628	53.863
	*a_RP_*	32.316	25.990	40.492	6.878	1.464
	*b_RP_*	0.406	0.837	0.831	0.960	1.073
	*R* ^2^	0.988	0.992	0.995	0.937	0.778
Tóth	*K_T_*	1.151	5.303	18.625	3.911	4.891
	α_*T*_	0.268	0.769	47.504	0.211	0.140
	*t*	96.680	9.875	0.881	8.454	4.937
	*R* ^2^	0.973	0.817	0.985	0.772	0.114
Fritz-Schlunder IV	β_1_	0.778	0.539	0.175	0.088	0.214
	β_2_	0.692	0.468	0.384	1.883	0.674
	α_1_	291.082	91.213	37.403	24.695	21.775
	α_2_	2.337	2.247	0.026	0.000	0.052
	*R* ^2^	0.980	0.954	0.971	0.851	0.914

## Supplementary Materials

The following are available online at https://www.mdpi.com/article/10.3390/ma14133738/s1, Figure S1: XRD patterns of oriented raw vermiculite-kaolinite clay (VK). k kaolinite, m muscovite, v vermiculite, q quartz, Figure S2: XRD patterns of FAU zeolites from VK for hydrothermal treatment (HT) times from 0 h to 72 h. Figure S3: XRD patterns FAU zeolites prepared using (a) fused sample to deionised (DI) water mass ratios of 1:5 to 1:15 and (b) different temperatures and times during the alkaline fusion step. Table S1: XRF chemical compositions (wt%) of reference zeolite (ZRef-FAU) and the optimal zeolite from clay (ZVK-FAU). Figure S4: Raw VK adsorption studies using 5 g L^−1^ clay loading for *C*_0_ = 200 mg L^−1^ and duration of 10 to 180 min.

## References

[1] Khalifa A.Z., Cizer Ö., Pontikes Y., Heath A., Patureau P., Bernal S.A., Marsh A.T.M. (2020). Advances in alkali-activation of clay minerals. Cem. Concr. Res..

[2] Abe S.S., Masunaga T., Honna T., Wakatsuki T. (2006). Comprehensive assessment of the clay mineralogical composition of lowland soils in West Africa. Soil Sci. Plant Nutr..

[3] Malamis S., Katsou E. (2013). A review on zinc and nickel adsorption on natural and modified zeolite, bentonite and vermiculite: Examination of process parameters, kinetics and isotherms. J. Hazard. Mater..

[4] Vhahangwele M., Mugera G.W. (2015). The potential of ball-milled South African bentonite clay for attenuation of heavy metals from acidic wastewaters: Simultaneous sorption of Co^2+^, Cu^2+^, Ni^2+^, Pb^2+^, and Zn^2+^ ions. J. Environ. Chem. Eng..

[5] Raghuvir P.B., Prabhu P.P., Prabhu B., Mathew T.M. (2018). A review on removal of heavy metal ions from waste water using natural/modified bentonite. MATEC Web Conf..

[6] Vega J.L., Ayala J., Loredo J., Iglesias J.G. Bentonites as adsorbents of heavy metals ions from mine waste leachates: Experimental data. Proceedings of the 9th International Mine Water Congress.

[7] Sen Gupta S., Bhattacharyya K.G. (2008). Immobilization of Pb(II), Cd(Ii) and Ni(II) ions on kaolinite and montmorillonite surfaces from aqueous medium. J. Environ. Manag..

[8] Sen Gupta S., Bhattacharyya K.G. (2012). Adsorption of heavy metals on kaolinite and montmorillonite: A review. Phys. Chem. Chem. Phys..

[9] Yavuz O., Altunkaynak Y., Guzel F. (2003). Removal of copper, nickel, cobalt and manganese from aqueous solution by kaolinite. Water Res..

[10] de Pablo L., Chávez M.L., Abatal M. (2011). Adsorption of heavy metals in acid to alkaline environments by montmorillonite and ca-montmorillonite. Chem. Eng. J..

[11] Matusik J., Wścisło A. (2014). Enhanced heavy metal adsorption on functionalized nanotubular halloysite interlayer grafted with aminoalcohols. Appl. Clay Sci..

[12] Anastopoulos I., Mittal A., Usman M., Mittal J., Yu G., Núñez-Delgado A., Kornaros M. (2018). A review on halloysite-based adsorbents to remove pollutants in water and wastewater. J. Mol. Liq..

[13] Yadav V.B., Gadi R., Kalra S. (2019). Clay based nanocomposites for removal of heavy metals from water: A review. J. Environ. Manag..

[14] Xu X., Cheng Y., Wu X., Fan P., Song R. (2020). La(III)-bentonite/chitosan composite: A new type adsorbent for rapid removal of phosphate from water bodies. Appl. Clay Sci..

[15] Payra P., Dutta P.K. (2004). Zeolites: A primer. ChemInform.

[16] Dyer A., Robson H., Lillerud K.P. (2001). Ion exchange capacity. Verified Syntheses of Zeolitic Materials.

[17] Komvokis V., Tan L.X.L., Clough M., Pan S.S., Yilmaz B. (2016). Zeolites in fluid catalytic cracking (FCC). Zeolites in Sustainable Chemistry.

[18] Asghari M., Mohammadi T., Aziznia A., Danayi M.R., Moosavi S.H., Alamdari R.F., Agand F. (2008). Preparation and characterization of a thin continuous faujasite membrane on tubular porous mullite support. Desalination.

[19] Cundy C.S., Cox P.A. (2003). The hydrothermal synthesis of zeolites: History and development from the earliest days to the present time. Chem. Rev..

[20] Ltaief O.O., Siffert S., Poupin C., Fourmentin S., Benzina M. (2015). Optimal synthesis of faujasite-type zeolites with a hierarchical porosity from natural clay. Eur. J. Inorg. Chem..

[21] Moneim M.A., Ahmed E.A. (2015). Synthesis of faujasite from Egyptian clays: Characterizations and removal of heavy metals. Geomaterials.

[22] Stamires D.N. (1973). Properties of the zeolite, faujasite, substitutional series: A review with new data. Clays Clay Miner..

[23] Ltaief O.O., Siffert S., Fourmentin S., Benzina M. (2015). Synthesis of faujasite type zeolite from low grade Tunisian clay for the removal of heavy metals from aqueous waste by batch process: Kinetic and equilibrium study. Comptes Rendus Chim..

[24] Belviso C., Cavalcante F., Niceforo G., Lettino A. (2017). Sodalite, faujasite and a-type zeolite from 2:1dioctahedral and 2:1:1 trioctahedral clay minerals. A singular review of synthesis methods through laboratory trials at a low incubation temperature. Powder Technol..

[25] Doyle A.M., Albayati T.M., Abbas A.S., Alismaeel Z.T. (2016). Biodiesel production by esterification of oleic acid over zeolite y prepared from kaolin. Renew. Energy.

[26] Krongkrachang P., Thungngern P., Asawaworarit P., Houngkamhang N., Eiad-Ua A. (2019). Synthesis of zeolite Y from kaolin via hydrothermal method. Mater. Today Proc..

[27] Hunter J.M. (1973). Geophagy in Africa and in the united states: A culture-nutrition hypothesis. Geogr. Rev..

[28] Chen X. (2015). Modeling of experimental adsorption isotherm data. Information.

[29] Ho Y.S., Porter J.F., McKay G. (2002). Equilibrium isotherm studies for the sorption of divalent metal ions onto peat: Copper, nickel and lead single component systems. Water Air Soil Pollut..

[30] Langmuir I. (2002). The constitution and fundamental properties of solids and liquids. Part I. Solids. J. Am. Chem. Soc..

[31] Foo K.Y., Hameed B.H. (2010). Insights into the modeling of adsorption isotherm systems. Chem. Eng. J..

[32] Freundlich H.M.F. (1906). Over the adsorption in solution. J. Phys. Chem..

[33] Toth J. (2000). Calculation of the bet-compatible surface area from any type I isotherms measured above the critical temperature. J. Colloid Interface Sci..

[34] Toth J. (1971). State equations of solid-gas interface layers. Acta Chim. Acad. Sci. Hung..

[35] Hamdaoui O., Naffrechoux E. (2007). Modeling of adsorption isotherms of phenol and chlorophenols onto granular activated carbon. Part II. Models with more than two parameters. J. Hazard. Mater..

[36] Al-Ghouti M.A., Da’ana D.A. (2020). Guidelines for the use and interpretation of adsorption isotherm models: A review. J. Hazard. Mater..

[37] Kumar S., Zafar M., Prajapati J.K., Kumar S., Kannepalli S. (2011). Modeling studies on simultaneous adsorption of phenol and resorcinol onto granular activated carbon from simulated aqueous solution. J. Hazard. Mater..

[38] Shigemoto N., Hayashi H., Miyaura K. (1993). Selective formation of na-x zeolite from coal fly ash by fusion with sodium hydroxide prior to hydrothermal reaction. J. Mater. Sci..

[39] Joseph I.V., Tosheva L., Doyle A.M. (2020). Simultaneous removal of Cd(II), Co(II), Cu(II), Pb(II), and Zn(II) ions from aqueous solutions via adsorption on FAU-type zeolites prepared from coal fly ash. J. Environ. Chem. Eng..

[40] Valtchev V.P., Bozhilov K.N. (2004). Transmission electron microscopy study of the formation of FAU-type zeolite at room temperature. J. Phys. Chem. B.

[41] Tosheva L., Brockbank A., Mihailova B., Sutula J., Ludwig J., Potgieter H., Verran J. (2012). Micron- and nanosized FAU-type zeolites from fly ash for antibacterial applications. J. Mater. Chem..

[42] Murayama N., Yamamoto H., Shibata J. (2002). Mechanism of zeolite synthesis from coal fly ash by alkali hydrothermal reaction. Int. J. Miner. Process..

[43] Berkgaut V., Singer A. (1996). High capacity cation exchanger by hydrothermal zeolitization of coal fly ash. Appl. Clay Sci..

[44] Mora-Fonz M.J., Catlow C.R., Lewis D.W. (2008). H-bond interactions between silicates and water during zeolite pre-nucleation. Phys. Chem. Chem. Phys..

[45] Wu Q., Meng X., Gao X., Xiao F.S. (2018). Solvent-free synthesis of zeolites: Mechanism and utility. Acc. Chem. Res..

[46] Bushuev Y.G., Sastre G., de Julián-Ortiz J.V. (2009). The structural directing role of water and hydroxyl groups in the synthesis of beta zeolite polymorphs. J. Phys. Chem. C.

[47] Mahamadi C., Almomani F. (2019). On the dominance of Pb during competitive biosorption from multi-metal systems: A review. Cogent Environ. Sci..

[48] Selim H.M. (2016). Competitive Sorption and Transport of Heavy Metals in Soils and Geological Media.

[49] Nebaghe K.C., El Boundati Y., Ziat K., Naji A., Rghioui L., Saidi M. (2016). Comparison of linear and non-linear method for determination of optimum equilibrium isotherm for adsorption of copper(II) onto treated Martil sand. Fluid Phase Equilib..

[50] Zand A.D., Abyaneh M.R. (2020). Adsorption of lead, manganese, and copper onto biochar in landfill leachate: Implication of non-linear regression analysis. Sustain. Environ. Res..

