# Supplementary material for: FAU-Type Zeolite Synthesis from Clays and Its Use for the Simultaneous Adsorption of Five Divalent Metals from Aqueous Solutions
Source: Materials (Basel). 2021 Jul 3;14(13):3738. doi: 10.3390/ma14133738 (PMC8269868; doi:10.3390/ma14133738)
Supplement: Supplementary file 1 [file materials-14-03738-s001.zip › materials-1222774-SM-updated.pdf]

materials

Article

FAU-Type Zeolite Synthesis from Clays and Its Use for the Simultaneous Adsorption of Five Divalent Metals from Aqueous Solutions

Ifeoma V. Joseph *, Lubomira Tosheva *, Gary Miller and Aidan M. Doyle

Department of Natural Sciences, Manchester Metropolitan University, Chester Street, Manchester M1 5GD, UK; g.miller@mmu.ac.uk (G.M.); a.m.doyle@mmu.ac.uk (A.M.D.)
* Correspondence: i.joseph@mmu.ac.uk (I.V.J.); l.tosheva@mmu.ac.uk (L.T.)

Citation: Joseph, I.V.; Tosheva, L.; Miller, G.; Doyle, A.M. FAU-Type Zeolite Synthesis from Clays and Its Use for the Simultaneous Adsorption of Five Divalent Metals from Aqueous Solutions. *Materials* **2021**, *14*, 3738. https://doi.org/10.3390/ma14133738

Academic Editors: Miguel A. Camblor and Serena Esposito

Received:29 April 2021
Accepted:30 June 2021
Published: 3 July 2021

Publisher's Note: MDPI stays neutral with regard to jurisdictional claims in published maps and institutional affiliations.

Copyright: © 2021 by the authors. Submitted for possible open access publication under the terms and conditions of the Creative Commons Attribution (CC BY) license (http://creativecommons.org/licenses/by/4.0/).

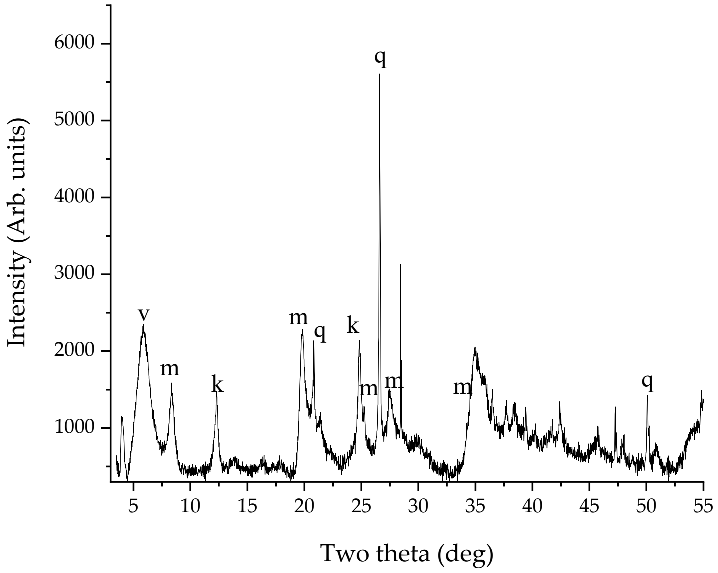

Figure S1. XRD patterns of oriented raw vermiculite - kaolinite clay (VK). k kaolinite, m muscovite, v vermiculite, q quartz.

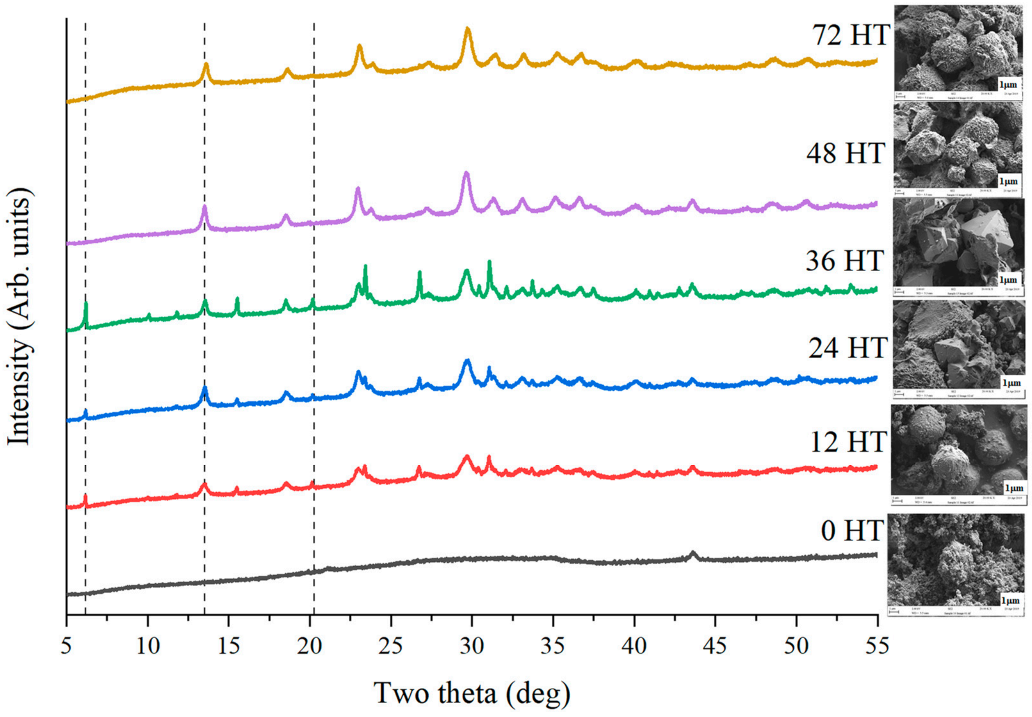

Figure S2. XRD patterns of FAU zeolites from VK for hydrothermal treatment (HT) times from 0 h to 72 h. Dashed lines represent the main FAU zeolite peaks. Inserts contain SEM images of the corresponding samples.

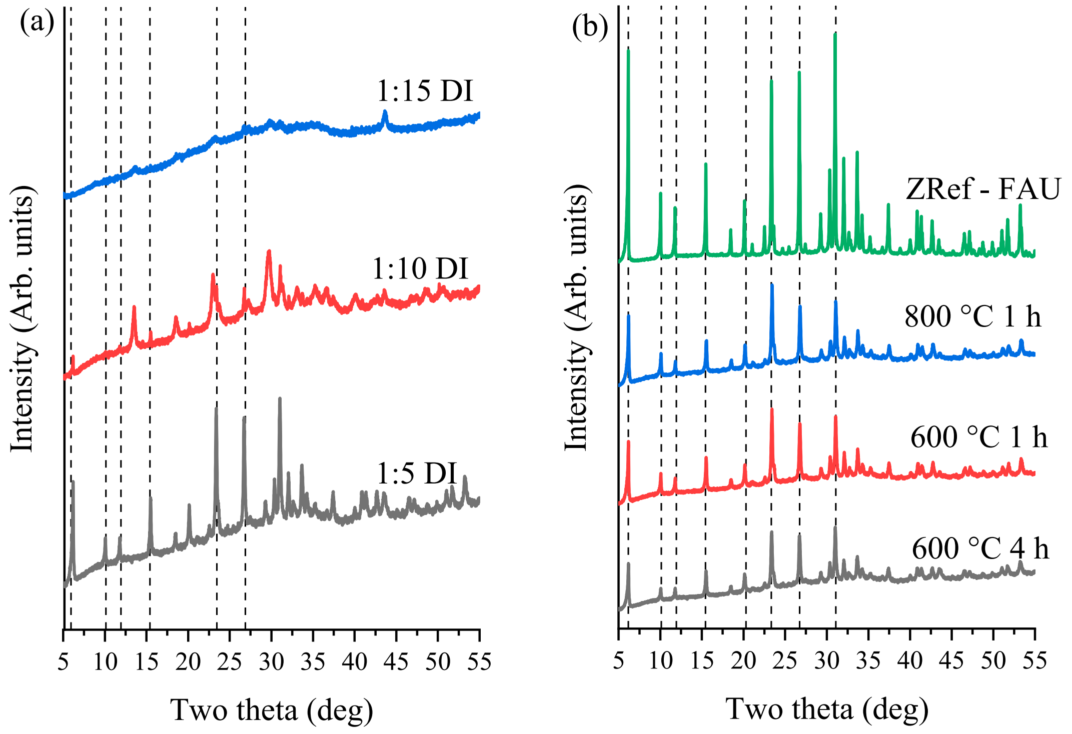

Figure S3. XRD patterns FAU zeolites prepared using (**a**) fused sample to deionised (DI) water mass ratios of 1:5 to 1:15 and (**b**) different temperatures and times during the alkaline fusion step. Dashed lines represent main FAU zeolite peaks.

Table S1. XRF chemical compositions (wt%) of reference zeolite (ZRef-FAU) and the optimal zeolite from clay (ZVK-FAU).

Adsorbent	SiO_2	Al_2O_3	Na_2O	MgO	Fe_2O_3	TiO_2	CaO
**ZRef-FAU**	32.20	23.50	18.90		0.03	0.00	0.01
**ZVK-FAU**	42.80	22.50	19.00	2.69	9.31	1.44	1.13

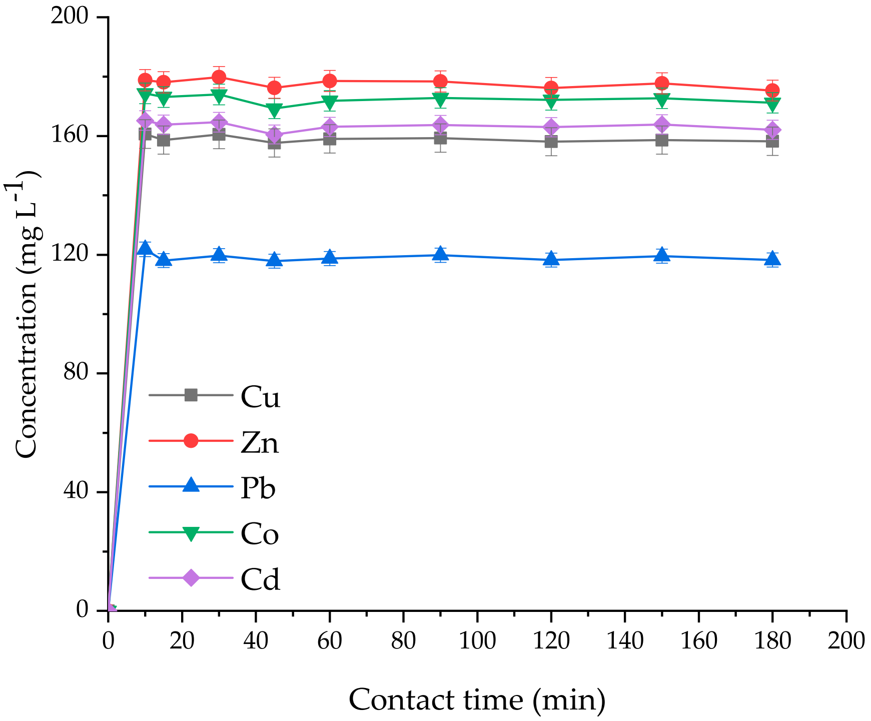

Figure S4. Raw VK adsorption studies using 5 g L^{-1} clay loading for C_0 = 200 mg L^{-1} and duration of 10 to 180 min.
